# Association of *IFNA16* and *TNFRSF19* Polymorphisms with Intramuscular Fat Content and Fatty Acid Composition in Pigs

**DOI:** 10.3390/biology11010109

**Published:** 2022-01-10

**Authors:** Supamit Mekchay, Nanthana Pothakam, Worrarak Norseeda, Pantaporn Supakankul, Tawatchai Teltathum, Guisheng Liu, Watcharapong Naraballobh, Trisadee Khamlor, Korawan Sringarm, Patcharin Krutmuang

**Affiliations:** 1 Department of Animal and Aquatic Sciences, Faculty of Agriculture, Chiang Mai University, Chiang Mai 50200, Thailand; Nanthana_Pothakam@cmu.ac.th (N.P.); watcharapong.n@cmu.ac.th (W.N.); trisadee.kha@cmu.ac.th (T.K.); korawan.s@cmu.ac.th (K.S.); 2 Center of Excellence on Agricultural Biotechnology: (AG-BIO/MHESI), Bangkok 10900, Thailand; 3 Cluster of Research and Development of Pharmaceutical and Natural Products Innovation for Human or Animal, Chiang Mai University, Chiang Mai 50200, Thailand; 4 Innovative Agriculture Research Center, Faculty of Agriculture, Chiang Mai University, Chiang Mai 50200, Thailand; patcharin.k@cmu.ac.th; 5 Veterinary, Conservation and Research Section, Animal Management Division, Chiang Mai Night Safari, Chiang Mai 50230, Thailand; 6 Department of Agriculture, Faculty of Agricultural Technology, Lampang Rajabhat University, Lampang 52100, Thailand; worrarak@live.lpru.ac.th; 7 Division of Animal Science, School of Agriculture and Natural Resources, University of Phayao, Phayao 56000, Thailand; pantaporn.su@up.ac.th; 8 Mae Hong Son Livestock Research and Breeding Center, Mae Hong Son 58000, Thailand; tawatchai2522@hotmail.com; 9 Institute of Animal Science and Veterinary Medicine, Hubei Academy of Agricultural Sciences, Wuhan 430064, China; guisheng_liu1964@yahoo.com; 10 Hubei Key Lab for Animal Embryo Engineering and Molecular Breeding, Wuhan 430064, China; 11 Department of Entomology and Plant Pathology, Faculty of Agriculture, Chiang Mai University, Chiang Mai 50200, Thailand

**Keywords:** fatty acid, *IFNA16*, intramuscular fat, MUFA, pork, SFA, *TNFRSF19*

## Abstract

**Simple Summary:**

Interferon-alpha-16 (IFNA16) and tumor necrosis factor receptor superfamily member 19 (TNFRSF19) are cytokines that may play a role in adipogenesis, lipid accumulation and fatty acid metabolism in the muscle tissue of mammals. However, no association study of the porcine *IFNA16* and *TNFRSF19* genes with the fatty acid composition of intramuscular fat has been reported in pigs. Therefore, the current study was performed to verify single nucleotide polymorphisms of the porcine *IFNA16* and *TNFRSF19* genes and to analyze how they affect intramuscular fat content and fatty acid composition in commercial crossbred pigs. The porcine *IFNA16* c.413G > A polymorphism was associated with stearic acid, total saturated fatty acids and the ratio of monounsaturated fatty acids to saturated fatty acids. Moreover, the *TNFRSF19* c.860G > C polymorphism was associated with intramuscular fat content and arachidic acid levels. The results revealed that the porcine *IFNA16* and *TNFRSF19* polymorphisms are related to lipid deposition and/or fatty acid composition in the muscle tissue of pigs. These findings suggest that the porcine *IFNA16* and *TNFRSF19* gene variants may contribute to lipid accumulation and fatty acid deposition in the muscle tissue of pigs.

**Abstract:**

Interferon-alpha-16 (IFNA16) and tumor necrosis factor receptor superfamily member 19 (TNFRSF19) are cytokines that may play a role in adipogenesis and fatness. Single nucleotide polymorphisms (SNPs) of the porcine *IFNA16* and *TNFRSF19* genes were verified and their association with intramuscular fat (IMF) content and fatty acid (FA) composition were evaluated in commercial crossbred pigs. Two non-synonymous SNPs of the porcine *IFNA16* c.413G > A and *TNFRSF19* c.860G > C loci were detected in commercial crossbred pigs. The porcine *IFNA16* c.413G >A polymorphism was significantly associated with stearic acid, total saturated FAs (SFAs), and the ratio of monounsaturated FAs (MUFAs) to SFAs (*p* < 0.05). Furthermore, the porcine *TNFRSF19* c.860G > C polymorphism was found to be significantly associated with IMF content and arachidic acid levels (*p* < 0.05). The results revealed that porcine *IFNA16* and *TNFRSF19* polymorphisms are related to IMF content and/or FA composition and affirmed the importance of these cytokine genes as potential candidate genes for lipid deposition and FA composition in the muscle tissue of pigs.

## 1. Introduction

Intramuscular fat (IMF) content is an important trait for the enhancement of meat quality in pigs [1,2]. Fatty acid (FA) composition has a strong impact on fat quality and is relevant to the nutrient value and edibility of pork [2,3,4]. Notably, high levels of FAs, especially oleic acid, palmitoleic acid, and total monounsaturated FAs (MUFAs) are known to be positively correlated with the flavor of pork and could be beneficial to human health [5]. Conversely, a high dietary intake of saturated FAs (SFAs) increases the risk of cardiovascular disease, diabetes, and lipid disorders in humans [5,6]. Therefore, reducing the content of SFAs while simultaneously enhancing the content of MUFAs in pork would be a fundamental achievement in the improvement of the genetic composition of intramuscular FAs [5]. Although it is known that IMF content and FA composition traits are genetically regulated with moderate heritability [7], the underlying physio-genetic complex mechanisms of these traits have not yet been clarified [8]. Several genome-wide association studies (GWAS) have been performed to identify genomic regions associated with IMF deposition and FA composition in pigs [4,5,9,10]. Additionally, the transcriptomic approaches have been applied to examine alterations in the transcription of numerous genes that are relevant to fatness and intramuscular FA composition in various pig breeds [11,12,13,14]. It has been shown that various cytokine genes are related to fatness and lipid metabolism in pigs. Recently, our previous study demonstrated that inflammatory adipocytokine *IL-1* and *IL-6* genes are associated with IMF content and FA composition in pigs [15]. Currently, numerous studies demonstrate that several inflammatory cytokines play an important role in lipid metabolism and are related to obesity in mammalian species [16,17,18]. Therefore, many inflammatory cytokines could be good candidate genes for fat deposition and lipid metabolism in muscles of pigs.

IFNA16 belongs to the type I interferon (IFN) alpha subtypes that are pleiotropic inflammatory cytokines possessing antiviral, antiproliferative, apoptotic, and immunoregulatory functions [19,20]. Numerous studies have demonstrated that IFN-alpha subtypes are involved in adipogenesis, lipogenesis, and lipid metabolism, as well as being related to obesity in humans [21,22,23,24]. The porcine *IFNA16* gene has been mapped on the *Sus scrofa* chromosome 1 (SSC1) at position 201.6 megabase pairs (Mb). The coding sequence of *IFNA16* is 570 base pairs (bp) in length. It is known to be composed of one exon and encodes a 189-amino acid peptide while possessing 294 SNPs in the gene (ENSSSCT00000035181; https://jul2019.archive.ensembl.org/index.html, accessed on 22 April 2021). The porcine *IFNA16* gene is located near the quantitative trait loci (QTL) regions for IMF content (184.6 Mb), stearic acid (210.6–293.0 Mb), and palmitoleic acid (210.6–252.3 Mb) [25,26].

TNFRSF19 is an orphan member of the tumor necrosis factor (TNF) receptor superfamily and is involved in cytokine and cytokine receptor interaction [27,28]. It binds with the TNF (inflammatory cytokine) ligand family member and is related to B-cell survival [28]. Moreover, *TNFRSF19* is a target of canonical Wnt signaling and adipogenic transcription factor CCAAT/enhancer-binding proteins (C/EBP). Moreover, it regulates mesenchymal stem cell (MSC) differentiation to either osteoblasts or adipocytes [29]. The porcine *TNFRSF19* gene has been mapped on SSC11 at position 2.5–2.6 Mb. Notably, the porcine *TNFRSF19* gene is located near the QTLs for IMF (1.9–4.3 Mb), oleic (5.2 Mb), and linoleic acid content (5.2–5.4 Mb) [30,31,32]. The coding sequence of *TNFRSF19* is 4970 bp in length. It consists of ten exons and nine introns and encodes a 418-amino acid peptide while possessing 5884 SNPs in the gene (ENSSSCT00000010168.4; https://asia.ensembl.org/index.html, accessed on 22 April 2021).

The above-mentioned information suggests that *IFNA16* and *TNFRSF19* genes may be related to lipid accumulation and FA content in the muscle tissue of mammals. However, despite their functional and positional candidacy, no association study of the porcine *IFNA16* and *TNFRSF19* genes has been reported in pigs with regards to IMF content and FA composition traits. Therefore, this study aimed to verify polymorphisms of the *IFNA16* and *TNFRSF19* genes and to elucidate their association with IMF content and FA composition in commercial crossbred pigs.

## 2. Materials and Methods

### 2.1. Animals and DNA Isolation

This study was conducted in the commercial crossbred Duroc and Large White × Landrace pigs (*n* = 478; 215 barrows and 263 gilts), as has been described in a previous study [15]. The pigs were fed the same corn–soybean diet that contained 3200 kcal/kg digestible energy and 16% crude protein and were maintained under the commercial environmental conditions outlined by the Betagro Group Company (Nakhon Ratchasima, Thailand). The slaughtering of these pigs was performed in a commercial abattoir following standard procedures (animal stunning was carried out using an electric stunner prior to slaughtering) for pigs that weighed about 90 kg. *Longissimus thoracis* (LT) muscle tissue samples taken from the 10th to 11th rib on the left side of each carcass were collected to assess IMF content and FA composition. Thus, ethical approval for this investigation was not a requirement. Genomic DNA samples were extracted from the LT muscle tissue samples by applying the phenol and chloroform protocol as described in a previous study [33] and were stored at 4 °C for further analysis. Concentrations of DNA samples were determined with a Nanodrop 2000c spectrophotometer (Thermo Scientific, Wilmington, DE, USA).

### 2.2. Phenotype Measurements

For IMF analysis, the tissue samples of the LT muscle (30 g) were freeze-dried and pulverized. Thereafter, an examination of the IMF content of LT muscle tissue samples was conducted using the ether extraction method according to the Association of Official Analytical Chemists regulations [34]. The expression of IMF content was reported as g of lipid in 100 g of muscle tissue. After the extraction of lipids, they were converted into FA methyl esters (FAMEs) using the method described in a previously published study [5]. The FA composition was evaluated with the method established in our previous study [15] by using a gas chromatography-flame ionization detector (GC-FID, SCION 456-GC, Bruker Daltonics, Fremont, CA, USA). Separation was performed on an RT-2560 capillary column (RESTEK, Bellefonte, PA, USA). The FAMEs were identified and quantified with a 37-component standard FAME Mix (RESTEK). Individually, FA composition values were reported as g per 100 g of total FAs. Subsequently, sums of SFAs (C12:0 + C14:0 + C16:0 + C18:0 + C20:0), MUFAs (C16:1n-7 + C18:1n-9 + C20:1n-9), and n-6 polyunsaturated FAs (PUFAs) (C18:2n-6 + C18:3n-6 + C20:2n-6 + C20:3n-6 + C20:4n-6) were estimated. Lastly, ratios of MUFAs to SFAs, MUFAs to n-6 PUFAs, and n-6 PUFAs to SFAs were then calculated.

### 2.3. Genotyping

To genotype the SNPs in the porcine *IFNA16* and *TNFRSF19* genes, five non-synonymous SNPs were selected based on the restriction enzymes available in the Ensembl database (ENSSSCT00000035181 and ENSSSCT00000010168.4). These five SNPs consisted of *IFNA16* c.199G > C (rs786793899), *IFNA16* c.413G > A (rs701706389), *TNFRSF19* c.250T > A (rs790893091), *TNFRSF19* c.860G > C (rs326658865), and *TNFRSF19* c.1188C > G (rs792463498). Primers were designed from the porcine *IFNA16* and *TNFRSF19* nucleotide sequences (GenBank accession number: NC_010443.5 and NC_010453.5, respectively) as is shown in Table 1. The SNPs of the porcine *IFNA16* and *TNFRSF19* genes were genotyped by using polymerase chain reaction–fragment length polymorphism (PCR-RFLP) assay. PCR amplifications were performed in 20 µL volumes consisting of 50 ng of genomic DNA sample, 1 × PCR (NH_4_)_2_SO_4_ buffer, 0.2 mM dNTPs, 0.4 mM each primer (Table 1), 1.5 mM MgCl_2_, and 0.2 U *Taq* DNA polymerase (Thermo Scientific, Hanover, MD, USA). The PCR was performed using an initial denaturing at 95 °C for 3 min followed by 32 cycles of 95 °C for 30 s, 58 to 60 °C for 30 s, and 72 °C for 30 s, and then 5 min at 72 °C to complete the reaction. The PCR products of the porcine *IFNA16* and *TNFRSF19* genes were digested with restriction enzymes (Thermo Scientific) as is shown in Table 1. The digested PCR products were electrophoresed on 6% polyacrylamide gels and stained with ethidium bromide for visualization in the gel documentation system.

### 2.4. Statistical Analysis

The genotype and allele frequencies were calculated for each SNP locus. A chi-square test was performed to examine the populations for Hardy–Weinberg equilibrium (HWE) and loci were considered in equilibrium for *p* values of the test > 0.05. Analysis of the effects of porcine *IFNA16* and *TNFRSF19* polymorphisms on IMF content and FA composition traits was conducted with the use of the general linear model as follows: Y*_ijk_ =* µ *+* S*_i_* + G*_j_ +* e*_ijk_*, where Y*_ijk_* is the phenotype values, μ is the overall mean value for each trait, S*_i_* is the fixed effect of sexes (*i* = 1–2), G*_j_* is the fixed effect of the *IFNA16* or *TNFRSF19* genotypes (*j* = 1–2 or 1–3), and e*_ijk_* is the residual error. In this study, sire and dam information were unavailable hence, they were not included in the statistical model. Lastly, the least square mean values between genotype groups for each locus were compared using the least significance differences (LSD) test (*p* < 0.05).

## 3. Results

### 3.1. Porcine IFNA16 and TNFRSF19 Polymorphisms

The PCR-RFLP patterns of five SNPs loci of the porcine *IFNA16* and *TNFRSF19* genes are presented in Table 1. Two SNPs of the porcine *IFNA16* c.413G > A and *TNFRSF19* c.860G > C loci were found to be segregated in this commercial pig population. The porcine *IFNA16* c.413G > A variant was a non-synonymous missense mutation in exon 1, leading to a non-conservative amino acid change at position 138 from glycine to aspartic acid (G138D). Moreover, the porcine *TNFRSF19* c.860G > C variant was also a non-synonymous missense mutation in exon 9 creating an amino acid change at position 287 from glycine to alanine (G287A). Additionally, three non-synonymous SNPs of porcine *IFNA16* c.199G > C (G67R), *TNFRSF19* c.250T > A (W84R), and *TNFRSF19* c.1188C > G (D396E) loci were positioned in exon 1, exon 4, and exon 9, respectively. However, these polymorphic loci were not found to be segregating in these commercial pigs. Thus, porcine *IFNA16* c.413G > A and *TNFRSF19* c.860G > C loci were used to study the association of their effects with IMF deposition and FA composition in muscle tissue samples taken from pigs.

### 3.2. Genotype and Allele Frequencies

All genotype and allele frequencies of the porcine *IFNA16* and *TNFRSF19* genes are presented in Table 2. Two genotypes (GG and GA) and two alleles (G and A) of the porcine *IFNA16* c.413G > A polymorphism were exhibited by these commercial crossbred pigs. No homozygous AA genotype of the porcine *IFNA16* c.413G > A polymorphism was observed in this study, whereas three genotypes (GG, GC, and CC) with two alleles (G and C) of the porcine *TNFRSF19* c.860G > C polymorphism were present among this pig population. The *IFNA16* c.413G and *TNFRSF19* c.860G alleles were determined to be more frequent in this commercial pig population. However, the three SNP markers of the porcine *IFNA16* c.199G > C, *TNFRSF19* c.250T > A, and *TNFRSF19* c.1188C > G loci were monomorphic, and their alleles were fixed as *IFNA16* c.199G, *TNFRSF19* c.250T, and *TNFRSF19* c.1188C among these crossbred pigs (data not shown). Moreover, there was a significant deviation in the genotype frequencies of the porcine *IFNA16* c.413G > A and *TNFRSF19* c.860G > C loci from the HWE (Table 2).

### 3.3. Association Analysis

The associations of the porcine *IFNA16* c.413G > A and *TNFRSF19* c.860G > C polymorphisms with IMF content and FA composition are reported in Table 3 and Table 4. No association of the porcine *IFNA16* c.413G > A polymorphism with the IMF trait was observed in this study. However, the porcine *IFNA16* c.413G > A polymorphism was significantly associated with stearic acid and SFA levels. Pigs with the GG genotype had lower stearic acid and SFA levels than pigs with the GA genotype. Hence, the porcine *IFNA16* c.413G allele appears to be a beneficial allele for stearic acid and SFA levels in these pigs. Additionally, the porcine *IFNA16* c.413G > A polymorphism was significantly associated with the ratio of MUFAs to SFAs and showed a tendency to be associated with oleic acid (*p* = 0.09), eicosadienoic acid (*p* = 0.07), and MUFA levels (*p* = 0.09). The porcine *TNFRSF19* c.860G > C polymorphism was significantly associated with IMF content and arachidic acid levels. Pigs with the CC genotype had higher IMF content when compared to pigs with the GC and CC genotypes. Furthermore, pigs with the CC and GC genotypes had lower arachidic acid levels than pigs with the GG genotype. Thus, the porcine *TNFRSF19* c.860C allele appears to be a beneficial allele for IMF content and arachidic acid levels. Moreover, the porcine *TNFRSF19* c.860G > C polymorphism showed a tendency to be related to stearic acid levels (*p* = 0.09).

## 4. Discussion

In this study, we have verified the presence of porcine *IFNA16* and *TNFRSF19* polymorphisms and evaluated their associations with IMF deposition and FA content in LT muscle tissues of commercial pigs. Two non-synonymous SNPs of the porcine *IFNA16* c.413G > A and *TNFRSF19* c.860G > C loci were segregated in this pig population. The chi-square analysis displayed significant deviations from the HWE specifications of the porcine *IFNA16* c.413G > A and *TNFRSF19* c.860G > C loci. From this result, it can be assumed that there are associated effects of selective mating based on certain desirable production traits that may be linked to the porcine *IFNA16* c.413G > A locus in this commercial pig population. In addition, null alleles may be another reason for this pig population to have deviated from the HWE. Moreover, it can be presumed that there was an excess of the heterozygosity of the porcine *TNFRSF19* c.860G > C locus present in this pig population. This may have been due to the outcrossing of their parent lines, which could have resulted in these pigs to have deviated from the HWE specifications.

In this present study, the porcine *IFNA16* c.413G > A gene variant was associated with stearic acid, SFA sum, and the ratio of MUFAs to SFAs. The porcine *IFNA16* c.413G allele appears to be a beneficial allele for these FA content traits due to lower stearic acid and SFA levels when compared with the *IFNA16* c.413A allele. Notably, the porcine polymorphism *IFNA16* c.413G > A is a non-synonymous SNP and presents amino acid substitution G138D. Although the function of the porcine *IFNA16* G138D gene variant has not been characterized yet, our results indicate a significant association of this *IFNA16* variant with stearic acid and SFA levels in LT muscle tissue samples collected from the commercial pig population. Therefore, it could be assumed that the porcine *IFNA16* G138D amino acid variant might be associated with the causative SNPs that are known to have a strong effect on muscle FA composition.

There has been a limited amount of published literature on the role of the *IFNA16* gene in fat deposition and FA content in the muscle tissue of mammals. However, numerous previous studies have reported that recombinant human IFN-alpha A/D stimulates hepatic lipogenesis both in vivo and in vitro [22,35]. Conversely, it is known to stimulate lipolysis in cultured adipocytes [36] and to reduce the adipose cell size that is related to the increased apoptosis of adipocytes [37]. In addition, IFN-alpha 2 serum levels were found to be negatively correlated with intramuscular fat in obese patients [38]. However, the results of this study imply that the porcine *IFNA16* polymorphism appeared to be unaffected by IMF content, which affirmed that this porcine *IFNA16* polymorphism affected FA levels but not IMF deposition. The porcine *IFNA16* gene variant had effectively reduced SFAs, especially stearic acid levels, and increased the ratio of MUFAs to SFAs. Thus, this outcome could have contributed to the increased levels in the nutritional value of pork and could be advantageous to human health [5]. Moreover, the porcine *IFNA16* polymorphism showed a tendency to be related to oleic acid and MUFA levels. Oleic acid is a major MUFA and is present as the most abundant FA in pork [4]. Importantly, it is positively correlated with the eating quality of pork [3,39]. Oleic acid is converted from stearic acid by the stearoyl-CoA desaturase (SCD) enzyme [40]. A positive association of the porcine *IFNA16* polymorphism with the ratio of oleic acid to stearic acid (desaturation index: C18:1n-9/C18:0 as an indicator of SCD activity) was detected in these commercial crossbred pigs (data not shown). This result indicates that the porcine *IFNA16* gene variant may be correlated with the SCD activity for MUFA synthesis. A previous study has demonstrated that IFN-alpha regulated the expression levels of the *SCD* gene in the liver of primates [41], while it may have also enhanced the SCD activity in patients with acute hepatitis C [42]. In addition, the genetic variation in the porcine *SCD* gene has been associated with MUFA content and the ratio of oleic acid to stearic acid in pork [4,6]. Moreover, a significantly negative correlation of oleic acid levels with stearic acid levels (r = −0.58, *p* < 0.001) was observed in this study. This result agreed with those of previous studies which found that the MUFA levels were negatively correlated with SFA levels [15]. The results of this study imply that *IFNA16* may be related to the lipid metabolism of SFAs and MUFAs, which would then increase the MUFA/SFA ratio. This evidence indicates that the *IFNA16* gene may be implicated in FA composition, especially with regard to SFA and MUFA levels.

In addition, an analysis of porcine *TNFRSF19* c.860G > C polymorphism indicated a strong association with IMF content and arachidic acid levels. The porcine *TNFRSF19* c.860C allele revealed higher IMF content than the porcine *TNFRSF19* c.860G allele. On another hand, the porcine *TNFRSF19* c.860C allele presented lower arachidic acid levels than the porcine *TNFRSF19* c.860G allele. Thus, the porcine *TNFRSF19* c.860C allele appears to be a beneficial allele for IMF content and arachidic acid levels. Porcine polymorphism *TNFRSF19* c.860G > C is a non-synonymous SNP and presents amino acid substitution G287A. Although the function of the porcine *TNFRSF19* G287A gene variant has not been characterized, the results indicate a significant association of the *TNFRSF19* variant with IMF content and arachidic acid levels. Therefore, it could be assumed that the change of this porcine *TNFRSF19* G287A amino acid might be related to the causative SNPs that have a strong effect on IMF content and FA composition.

Despite the fact that the knowledge of the function of *TNFRSF19* in fat deposition and FA content in the muscle tissue of mammals remains scant, the results of this study imply that the porcine *TNFRSF19* gene did have an effect on IMF deposition. A strong relationship between *TNFRSF* gene members and adipogenesis has been reported in various animals [29,43,44]. The *TNFRSF19* gene expression in human MSCs was determined to be inhibited by C/EBP and related to adipogenesis [29]. Moreover, the transcriptional analysis revealed that the *TNFRSF19* gene was downregulated in the adipose tissue of obese poultry [44,45]. In addition, a previous study has reported on the negative correlation of *TNFRSF6* expression levels and IMF content in Korean Hanwoo cattle [43]. Thus, the suppression of *TNFRSF19* and *TNFRSF6* genes seems to promote adipogenesis and fatness in animals. Conversely, the other *TNFRSF* gene members (e.g., *TNFRSF4*, *TNFRSF9*, *TNFRSF14*) were observed to be positively correlated with obesity in mammalian species [46,47,48]. The above-referenced evidence indicates that *TNFRSF19* plays a major role in adipogenesis by regulating the C/EBP transcription factor and may be important for IMF deposition. Moreover, the porcine *TNFRSF19* polymorphism had an effective impact on arachidic acid levels and exhibited a tendency to be related to stearic acid levels. Stearic acid is a precursor for the de novo biosynthesis of arachidic and oleic acids [40,49]. The porcine *TNFRSF19* polymorphism was significantly associated with the ratio of arachidic to stearic acid levels but was not associated with the ratio of oleic to stearic acid levels (data not shown). This result indicates that the porcine *TNFRSF19* gene may be correlated with FA synthetase (FASN, a key enzyme that catalyzes the de novo biosynthesis of SFAs) but it was not related to the SCD enzyme activity. Several studies have demonstrated that the polymorphism of the porcine *FASN* gene is associated with arachidic acid levels in the pork loin and backfat tissues [40,50]. It has been hypothesized that arachidic acid accumulation in the porcine adipocyte is the result of plasma FA uptake [40]. In this study, stearic acid showed a positive phenotypic correlation with arachidic acid levels (data not shown). This result agreed with previous studies, which indicated that stearic acid had a positive phenotypic and genetic correlation with arachidic acid levels in the backfat tissue of pigs [49,50]. Moreover, numerous previous studies have proposed the elongation of long-chain fatty acid family number 7 (*ELOVL7)* as a strong candidate gene that is indicative of the GWAS signals associated with arachidic acid levels and the metabolic indexes of arachidic to stearic acid or the ratios of eicosenoic to arachidic acid levels [9,10,51,52]. The evidence presented above indicates that the *TNFRSF19* gene may have a relationship with arachidic acid levels and may be related to the genes that regulate FA metabolism in muscle tissues. Therefore, the results of this study imply that the *TNFRSF19* gene may contribute to IMF deposition and be related to the FA metabolic pathway of arachidic acid.

With all of the above-mentioned evidence, it can be hypothesized that porcine *IFNA16* and *TNFRSF19* genes may play a crucial role in lipid accumulation and are relevant to FA metabolism in muscle tissue. The results of this study indicate that the porcine *IFNA16* and *TNFRSF19* genes could be expected to be involved in IMF content and FA composition. However, the effects of the *IFNA16* and *TNFRSF19* genes on IMF deposition and FA composition would need to be affirmed in larger pig populations. Moreover, further studies are required to better understand the molecular mechanisms of the *IFNA16* and *TNFRSF19* genes in regulating fat deposition and FA composition in the muscles of pigs. Furthermore, the relationship between *IFNA16* and *TNFRSF19* genes and metabolic FA enzymes should be identified in order to explain the specific pathways of intramuscular FA metabolism. These elucidations will enhance our knowledge on the effect of these cytokine genes on lipid content and FA composition. This determination could be significantly beneficial, not only for agricultural animals but also for biomedical research on human obesity.

## 5. Conclusions

In this current study, we validated SNPs in the porcine *IFNA16* and *TNFRSF19* genes and analyzed their association with IMF content and FA composition in LT muscle tissues of pigs. The porcine *IFNA16* polymorphism had a clear effect on stearic acid, total SFAs, and the ratio of MUFAs to SFAs. Moreover, the porcine *TNFRSF19* polymorphism revealed an association with IMF content and arachidic acid levels. Additionally, the favorable alleles of porcine *IFNA16* c.413G and *TNFRSF19* c.860C alleles were found to be beneficial for improving the stearic acid, total SFAs, the ratio of MUFAs to SFAs and IMF content in pork. Therefore, these favorable alleles may be used as marker-assisted selection to improve the quality of pork. These findings highlight the significance of the porcine *IFNA16* and *TNFRSF19* genes in IMF content and/or FA composition in pork. Thus, the porcine *IFNA16* and *TNFRSF19* genes may be potential candidate genes to enhance IMF content and FA composition in the muscles of pigs.

## Figures and Tables

**Table 1:** Primer sequences and restriction enzymes used to detect polymorphisms of porcine *IFNA16* and *TNFRSF19* genes and the pattern of PCR-RFLP fragment sizes; Ta, annealing temperature.

SNPs	Primer Sequence (5′ to 3′)	Size (bp)	Ta (°C)	Restriction Enzymes	PCR-RFLP Pattern (bp)
*IFNA16* c.199G > C	F: CTGAGGCTCCTGGCACAAAT R: TGAGCCTTCTGGACCTGGTT	113	60	*Sty*I	G: 83 + 30 C: 113
*IFNA16* c.413G > A	F: TTCTGCACTGGACTGGATCA R: GGAAGTATTTCCTCACAGCC	121	60	*Hin*fI	G: 121 A: 91 + 30
*TNFRSF19* c.250T > A	F: GCCGCACAGGTTCAAGGAG R: CGCTGGTGGCAGAGCAGTT	104	58	*Bse*NI	T: 78 + 26 A: 104
*TNFRSF19* c.860G > C	F: TCTGCCCACCCCCATACTAA R: CCGAGGAGGTCAGGGTAAGA	195	60	*Bsh*1236I	G: 116 + 79 C: 83 + 79 + 33
*TNFRSF19* c.1188C > G	F: ACACAGCTCGCTGCCAGAA R: GAGGCCTGTCTGGGGGCT	109	60	*Tai*I	C: 78 + 31 G: 109

**Table 2:** Genotype and allele frequencies of porcine *IFNA16* and *TNFRSF19* genes in pigs.

SNPs	*n*	Genotype Frequencies			Allele Frequencies ^1^		*p* Value ^2^ (χ^2^)
		AA	AB	BB	A	B	
*IFNA16* c.413G > A	468	0.67	0.33	0.00	0.83	0.17	<0.01 **
*TNFRSF19* c.860G > C	463	0.13	0.79	0.08	0.52	0.48	<0.01 **

^1^ Allele A represents major alleles of the porcine *IFNA16* c.413G and *TNFRSF19* c.860G loci and allele B represents minor alleles of the porcine *IFNA16* c.413A and *TNFRSF19* c.860C loci. ^2^ *p* value is considered a significant level of the chi-square (χ^2^) test for Hardy–Weinberg equilibrium of each locus, ** *p* < 0.01.

**Table 3:** Association of porcine *IFNA16* c.413G > A gene with IMF content and FA composition traits in *longissimus thoracis* muscles of pigs.

Traits	Genotypes (Least Squares Mean ± SE)		*p* Value
	GG (*n* = 313)	GA (*n* = 155)	
IMF	2.214 ± 0.267	2.168 ± 0.355	0.7014
Lauric acid (C12:0)	0.108 ± 0.006	0.113 ± 0.010	0.7269
Myristic acid (C14:0)	1.432 ± 0.085	1.549 ± 0.138	0.4818
Palmitic acid (C16:0)	15.515 ± 0.731	17.028 ± 1.192	0.2922
Stearic acid (C18:0)	12.369 ± 0.550 ^a^	15.177 ± 0.896 ^b^	0.0148
Arachidic acid (C20:0)	0.336 ± 0.044	0.363 ± 0.072	0.7494
SFAs	29.654 ± 1.166 ^a^	34.118 ± 1.899 ^b^	0.0490
Palmitoleic acid (C16:1n-7)	4.671 ± 0.255	4.638 ± 0.415	0.9468
Oleic acid (C18:1n-9)	39.737 ± 2.183	32.520 ± 3.556	0.0991
Eicosenoic acid (C20:1n-9)	2.192 ± 0.250	2.119 ± 0.407	0.8810
MUFAs	46.601 ± 2.201	39.278 ± 3.584	0.0971
Linoleic acid (C18:2n-6)	17.464 ± 1.498	19.864 ± 2.440	0.4121
γ-Linolenic acid (C18:3n-6)	0.096 ± 0.046	0.027 ± 0.076	0.4472
Eicosadienoic acid (C20:2n-6)	1.424 ± 0.167	2.016 ± 0.272	0.0791
Dihomo-γ-linolenic acid (C20:3n-6)	0.206 ± 0.036	0.303 ± 0.060	0.1878
Arachidonic acid (C20:4n-6)	0.062 ± 0.065	0.043 ± 0.027	0.6259
n-6 PUFAs	19.254 ± 1.594	22.211 ± 2.596	0.3436
MUFAs/SFAs	1.582 ± 0.079 ^b^	1.224 ± 0.137 ^a^	0.0360
MUFAs/n-6 PUFAs	2.392 ± 0.276	1.790 ± 0.602	0.5508
n-6 PUFAs/SFAs	0.679 ± 0.059	0.659 ± 0.101	0.8661

IMF: intramuscular fat content, MUFAs: monounsaturated fatty acids, n-6 PUFAs: n-6 polyunsaturated fatty acids, SFAs: saturated fatty acids. IMF is reported as g of lipid in 100 g of muscle tissue, while fatty acids (FAs) are reported as g of FA in 100 g of total FAs. Values in each row with different superscript letters are considered significantly different (^a,b^ *p* < 0.05).

**Table 4:** Association of porcine *TNFRSF19* c.860G > C gene with IMF content and FA composition traits in *longissimus thoracis* muscles of pigs.

Traits	Genotypes (Least Squares Mean ± SE)			*p* Value
	GG (*n* = 60)	GC (*n* = 365)	CC (*n* = 38)	
IMF	2.213 ± 0.285 ^a^	2.031 ± 0.381 ^a^	2.874 ± 0.545 ^b^	0.0258
Lauric acid (C12:0)	0.093 ± 0.016	0.099 ± 0.004	0.098 ± 0.007	0.9072
Myristic acid (C14:0)	1.244 ± 0.165	1.231 ± 0.045	1.247 ± 0.072	0.9770
Palmitic acid (C16:0)	14.129 ± 1.590	13.728 ± 0.434	14.108 ± 0.695	0.8643
Stearic acid (C18:0)	11.872 ± 1.279	11.501 ± 0.349	12.845 ± 0.559	0.0904
Arachidic acid (C20:0)	0.534 ± 0.101 ^b^	0.246 ± 0.027 ^a^	0.319 ± 0.044 ^a^	0.0111
SFAs	27.874 ± 2.341	26.806 ± 0.639	28.618 ± 1.024	0.2590
Palmitoleic acid (C16:1n-7)	4.551 ± 0.576	4.166 ± 0.157	4.197 ± 0.252	0.8004
Oleic acid (C18:1n-9)	33.925 ± 3.722	34.398 ± 1.016	33.503 ± 1.628	0.8769
Eicosenoic acid (C20:1n-9)	2.120 ± 0.550	1.983 ± 0.150	2.076 ± 0.240	0.9190
MUFAs	40.597 ± 3.864	40.549 ± 1.055	39.776 ± 1.69	0.9126
Linoleic acid (C18:2n-6)	22.857 ± 2.878	23.970 ± 0.785	21.380 ± 1.258	0.1678
γ-Linolenic acid (C18:3n-6)	0.082 ± 0.085	0.132 ± 0.023	0.127 ± 0.037	0.8445
Eicosadienoic acid (C20:2n-6)	1.895 ± 0.566	1.680 ± 0.154	1.608 ± 0.247	0.8845
Dihomo-γ-linolenic acid (C20:3n-6)	0.247 ± 0.114	0.196 ± 0.031	0.149 ± 0.049	0.5917
Arachidonic acid (C20:4n-6)	0.221 ± 0.156	0.224 ± 0.042	0.105 ± 0.068	0.2923
n-6 PUFAs	25.304 ± 2.893	26.003 ± 0.790	23.372 ± 1.265	0.1633
MUFAs/SFAs	1.497 ± 0.214	1.431 ± 0.102	1.388 ± 0.134	0.8405
MUFAs/n-6 PUFAs	1.587 ± 0.790	1.565 ± 0.534	1.682 ± 0.657	0.8895
n-6 PUFAs/SFAs	0.852 ± 0.132	0.826 ± 0.063	0.784 ± 0.083	0.1634

IMF: intramuscular fat content, MUFAs: monounsaturated fatty acids, n-6 PUFAs: n-6 polyunsaturated fatty acids, SFAs: saturated fatty acids. IMF is reported as g of lipid in 100 g of muscle tissue, while fatty acids (FAs) are reported as g of FA in 100 g of total FAs. Values in each row with different superscript letters are considered significantly different (^a,b^ *p* < 0.05).

## Data Availability

The data presented in this study are available on reasonable request from the corresponding author.

## Abbreviations

bp: base pair, C/EBP: CCAAT/enhancer-binding protein, ELOVL7: elongation of long-chain fatty acid family number 7, FA: fatty acid, FAME: fatty acid methyl ester, FASN: fatty acid synthetase, GC-FID: chromatography-flame ionization detector, GWAS: genome-wide association study, HWE: Hardy–Weinberg equilibrium, IFN: interferon, IFNA16: interferon-alpha-16, IMF: intramuscular fat, LT: *Longissimus thoracis*, Mb: megabase pair, MSC: mesenchymal stem cell, MUFA: monounsaturated fatty acid, PCR: polymerase chain reaction, PUFA: polyunsaturated fatty acid, QTL: quantitative trait loci, RFLP: fragment length polymorphism, SCD: stearoyl-CoA desaturase, SFA: saturated fatty acid, SNP: single nucleotide polymorphism, SSC: *Sus scrofa* chromosome, Ta: annealing temperature, TNF: tumor necrosis factor, TNFRSF19: tumor necrosis factor receptor superfamily member 19.
